# Willingness toward voluntary counseling and testing and associated factors among tuberculosis infected patients at public hospitals in Addis Ababa, Ethiopia

**DOI:** 10.3389/fpubh.2024.1354067

**Published:** 2024-08-06

**Authors:** Samuel Dessu Sifer, Milkiyas Solomon Getachew

**Affiliations:** ^1^Department of Public Health, Yekatit 12 Hospital Medical College, Addis Ababa, Ethiopia; ^2^Department of Public Health, College of Medicine and Health Sciences, University of Gondar, Gondar, Ethiopia

**Keywords:** willingness, VCT, TB, public hospitals, Addis Ababa

## Abstract

**Background:**

Voluntary counseling and testing for HIV has proven to be a highly effective and cost-efficient approach in many locations, yielding excellent results. It serves as a gateway to a range of HIV-related services, including the provision of antiretroviral drugs. Therefore, this study was aimed to assess the willingness toward VCT and associated factors among TB infected patients at Public Hospitals in Addis Ababa, Ethiopia; 2023.

**Methods:**

A facility-based cross-sectional study was undertaken at public hospitals in Addis Ababa from 1st to 30th of March 2023 with 235 participants using systematic random sampling. Trained data collectors employed a pretested data extraction tool for information gathering. Variables with *p*-value less than 0.05 in the multivariable logistic regression were considered statistically significant.

**Results:**

The prevalence of willingness toward VCT among TB infected patients was (78.3, 95%CI: 72.8, 83.4). Individuals with a primary education level (AOR: 6.32; 95%CI: 1.65, 24.25), government employees (AOR: 5.85; 95%CI: 1.78, 19.22) and private employees (AOR: 3.35; 95%CI: 1.12, 10.01), good knowledge of VCT (AOR: 3.12; 95%CI: 1.36, 7.16), perceived a higher risk (AOR: 6.58; 95%CI: 2.44, 17.73) and perceived stigma (AOR: 14.95; 95%CI: 4.98, 44.91) were factors associated with willingness toward VCT.

**Conclusion:**

The proportion of Tuberculosis infected patients expressing willingness toward Voluntary Counseling and Testing in this study was higher than in previous studies, it falls below the UNAIDS target of 90% of people knowing their HIV status. Notably, factors such as level of education, occupation, knowledge, perceived risk, and perceived stigma emerged as independent factors significantly associated with the willingness of TB-infected patients to undergo VCT. These findings underscore the importance of considering socio-demographic characteristics, knowledge levels, and psychosocial factors in designing strategies to enhance VCT acceptance among TB-infected individuals.

## Background

The World Health Organization (WHO) estimates that around one-third of the world’s population is infected with *Mycobacterium tuberculosis*, resulting in nearly 8.7 million incident cases of tuberculosis (TB) and approximately 1.4 million deaths (1). Notably, TB remains the leading cause of death among individuals living with HIV/AIDS (PLWHA) (2). These statistics underscore the pervasive impact of TB on a global scale and emphasize the critical association between TB and HIV/AIDS. Comprehensive strategies are necessary to address both diseases concurrently and effectively combat their public health implications.

The primary cause for the failure to achieve TB control targets in regions with a high prevalence of HIV is attributed to HIV infection itself. HIV infection is associated with a progressive decline in immune response and contributes to the pathogenesis of TB. This heightened susceptibility increases the risk of co-infection, leading to a more frequent occurrence of extra-pulmonary disease (3). Individuals infected with both HIV and TB face a higher annual risk of reactivating TB infection, estimated at 7–10%, compared to the 10% lifetime risk in those who are HIV-seronegative (4). To effectively address TB in regions with a high prevalence of HIV, the Stop TB strategy necessitates the implementation of collaborative TB/HIV activities within the country (3, 5). This collaborative approach is crucial for achieving successful control measures in the context of coexisting HIV and TB epidemics.

Globally, only 20% of individuals living with HIV are aware of their status, and just one in four people estimated to be co-infected with HIV and TB are identified and treated for both diseases (6). In response to this challenge, UNAIDS established the 90–90-90 targets in December 2013. These targets aim for 90% of people to be aware of their HIV status, with 90% of those individuals being connected to care, and ultimately, 90% achieving viral suppression (7). These targets set by UNAIDS reflect a comprehensive approach to improving awareness, access to care, and treatment outcomes for individuals affected by HIV, particularly those co-infected with TB.

The implementation of voluntary counseling and testing (VCT) for human immunodeficiency virus (HIV) has yielded excellent results in various locations. This approach is not only cost-effective but also serves as a gateway to accessing a range of HIV-related services, including the provision of antiretroviral drugs (8). As HIV/AIDS continues to spread globally, VCT have become integral components of efforts to prevent and control the impact of HIV (9). This underscores the significance of VCT as a pivotal strategy in the broader framework of HIV prevention and management initiatives.

In this approach, fundamental principles such as confidentiality, consent, and counseling remain crucial, and the standard pre-test counseling employed in VCT is adjusted to ensure informed consent without a comprehensive education and counseling session (10). In order to obtain informed consent, individuals must be aware of several key aspects: (i) the clinical and preventive advantages of testing; (ii) their right to refuse the test; (iii) the subsequent services that will be provided; and (iv) the significance of sharing test results with a partner in the event of a positive outcome (11). This emphasizes the importance of ensuring that individuals undergoing HIV testing are well-informed and empowered to make decisions regarding their healthcare.

Counseling and testing for HIV not only offer opportunities for preventing and treating HIV infections but also play a crucial role in the care of HIV patients. Moreover, during counseling and testing visits, there is a substantial demand for the co-management of TB-HIV cases (12). Obtaining information about the prevalence of HIV among TB patients is vital for addressing the growing demand for HIV/AIDS care and support, including the administration of antiretroviral therapy (ART), for individuals co-infected with HIV and TB (3). This underscores the importance of integrated approaches to address the dual challenges of TB and HIV within affected populations.

Conducting routine HIV testing for all patients in settings with generalized HIV epidemics is recommended due to the potential benefits of early diagnosis. This approach can help prevent morbidity, mortality, and sustained transmission by enabling the initiation of cotrimoxazole prophylaxis and timely antiretroviral treatment (ART) (13). Both the World Health Organization (WHO), the Joint United Nations Program on HIV/AIDS (UNAIDS), and the International Standards for TB Care endorse the provision of counseling services and HIV testing for every TB patient, particularly in countries with a high prevalence of HIV infection, exceeding 1% of the general population (14–16). This underscores the global recognition of the importance of integrated strategies in managing both HIV and TB for improved health outcomes (17, 18).

Presently, there is a lack of robust data on routine HIV testing and counseling of TB patients, highlighting the need for obtaining reliable information (19, 20). One suggested approach to address this issue is strengthening routine data through periodic surveys (21, 22). In numerous countries, the HIV prevalence among TB patients serves as a dependable indicator reflecting the spread of HIV into the general population (23–25). This study finding offer valuable insights for healthcare providers and policymakers to improve integrated TB and HIV care in Addis Ababa. Therefore, this study aimed to assess the willingness of TB patients toward VCT at Public Hospitals in Addis Ababa.

## Materials and methods

### Study design, area and period

A facility-based cross-sectional study was undertaken in the city of Addis Ababa, the capital of Ethiopia. Addis Ababa boasts a population of 2,738,248 million, experiencing an annual growth rate of 2.1%. The city is organized into 11 sub-cities and 99 kebeles, which represent the lowest administrative units within the urban structure. This study specifically focuses on 12 public hospitals situated in Addis Ababa, namely Tikur Anbessa Specialized Hospital, St. Peters Hospital, Alert Hospital, St. Paul Hospital Millennium Medical College, Zewditu Memorial, Yekatit 12, Minilik II, Ras Desta Damtew Hospital, Tirunesh Beijing Hospital, Dejazmach Balcha Hospital, Torhailoch Hospital, Police Hospital, and Gandhi Memorial Hospital. The study period spanned from the 1st to the 30th of March 2023.

### Populations

The source population for this study comprised all TB infected patients receiving care at public hospitals in Addis Ababa, while the study population encompassed a randomly selected subset of TB-infected patients at these public hospitals. All TB-infected patients in public hospitals in Addis Ababa were included in the study, with the exclusion criteria encompassing individuals who were unable to communicate effectively, those with severe illness, and pregnant women.

### Sample size determination and sampling procedure

The sample size for the first objective was determined using the single proportion formula with the following assumptions: a proportion level (“p”) of 73%, a 95% confidence level, and a 5% margin of error (degree of precision, “d”). The standard normal value at a 95% confidence level (Z ± α/2) was considered as 1.96. Applying these values to the formula, the initial sample size was calculated to be 303. Given that the average number of TB patients with follow-up in public hospitals in Addis Ababa is 710, and recognizing that the total source population is less than 10,000, a correction formula was applied, resulting in a sample size of 213. Incorporating a 10% adjustment for potential non-response, the final sample size was determined to be 235 (213 + 22).

The five governmental hospitals in Addis Ababa, namely Menelik II Referral Hospital, Ras Desta Hospital, St. Paul Hospital Millennium Medical College, Yekatit 12 Hospital Millennium Medical College, and St. Peter Hospital, were chosen through a simple random sampling method. The sample size was allocated proportionally to each selected hospital. Subsequently, study participants were chosen using a systematic sampling method, where the sampling fraction was determined by the formula *k* = *N*/*n*, resulting in *k* = 470/235 = 2. The initial sample was randomly selected, and subsequent samples were obtained by adding the sampling fraction of 2.

### Variables

The dependent variable for this study was willingness toward VCT and the independent variables were socio demographic factors (age, sex, residence, educational status, occupational status and estimated monthly income), self-perception (perceived risk, perceived stigma, perceived self-imagination and perceived support) and knowledge toward VCT.

### Operational definitions

#### Good knowledge

The mean of correct answers was computed and those who score a value greater than or equal to the mean were considered knowledgeable (26).

#### Poor knowledge

The mean of correct answers was computed and those who score a value lower than or equal to the mean were considered knowledgeable (26).

### Data collection tool and procedures

The data collection process involved face-to-face interviews utilizing pre-tested structured questionnaires. The questionnaire was developed after a thorough review of relevant literature, with necessary modifications (27–30). Initially prepared in English, it underwent translation to Amharic and subsequent translation back to English to ensure consistency. Six diploma nurses and two public health officers fluent in Amharic were recruited as data collectors and supervisors, respectively. The face-to-face interview technique was employed, following proper orientation for each participant regarding the study’s purpose and significance. Verbal consent was obtained before initiating the interviews, conducted at the participants’ residences.

### Data quality assurance

To ensure data quality, the data collection tool underwent a meticulous development process, involving an extensive review of relevant literature and similar studies (31–34). The well-designed instrument was translated into Amharic and provided to data collectors and supervisors after proper training. The training, conducted over 2 days by the principal investigator, covered the study’s objectives, procedures, data collection techniques, interviewing skills, and data management procedures. A pre-test of the questionnaire was conducted on 5% of the samples at Zewditu Memorial Hospital 1 week before the actual data collection period. Based on the pretest results, necessary corrections were implemented to enhance clarity and improve question completion. Throughout the data collection period, daily reviews and cross-checks were performed to ensure the completeness and relevance of the collected data before the data entry process.

### Data processing and analysis

Data coding, cleaning, and entry were carried out using Epi-data version 3.0.1, and the data were subsequently exported to the Statistical Package for the Social Sciences (SPSS) version 25.0. The dataset underwent additional recoding and cleaning procedures to address inconsistencies and missing values before the analysis. Descriptive statistics, including frequency distributions and mean values, were computed. The questionnaire included four questions with responses categorized as “Yes” or “No,” focusing on the study subjects’ knowledge about VCT.

Bivariate analysis was initially conducted to assess the association between independent variables and the dependent variable. Subsequently, both bivariate and multivariable logistic regression analyses were employed to identify factors associated with willingness toward VCT. The significance level was set at a *p*-value <0.25 with a 95% confidence interval (CI) for crude odds ratios (COR) during bivariate analysis and <0.05 with a 95% CI for adjusted odds ratios (AOR) during multivariable analysis. Finally, the study determined adjusted odds ratios (AOR) with 95% CI to identify factors significantly associated with willingness toward VCT, considering variables with a *p*-value <0.05 as statistically significant.

### Ethical considerations

The study adhered to pertinent guidelines and regulations throughout all procedures. Ethical clearance was obtained from the Yekatit 12 Hospital Medical College ethical review board. Prior to participation, all individuals provided informed verbal consent.

## Results

### Socio demographic characteristics

The study included a total of 235 participants, achieving a response rate of 100%. The participants’ ages ranged from 23 to 45 years, with a mean age of 33 ± 7 years. A majority of the participants (54.5%) were below the mean age, and most of them (73.6%) were males. Regarding education, the majority (40.9%) had attained college and above, while a small proportion (8.9%) had no formal education (Table 1). The minimum and maximum estimated monthly income of the study participants was 500.00ETB and 20000.00ETB, respectively, with the mean of 9165.00 ± 5411.00 ETB (Table 1).

**Table 1:** Socio demographic characteristics of TB infected patients at public hospitals in Addis Ababa, Ethiopia; 2023.

Variables	Category	Frequency (*n*)	Percentage (%)
Age (in years)	Below average	128	54.5
	More than average	107	45.5
Sex	Male	173	73.6
	Female	62	26.4
Level of education	No formal education	21	8.9
	Primary	54	23.0
	Secondary	64	27.2
	College and above	96	40.9
Occupation	Government employee	65	27.7
	Private employee	82	34.9
	Student	42	17.9
	Merchant	46	19.6

### Self-perception and related characteristics

In terms of self-perception, 160 (68.1%) of the study participants acknowledged a perceived risk of acquiring HIV infection. Additionally, 171 (72.8%) of the respondents had self-imagined scenarios related to HIV, and a substantial majority, 186 (79.1%), believed they would require support if diagnosed with HIV (Table 2).

**Table 2:** Self-perception and related characteristics of TB infected patients at public hospitals in Addis Ababa, Ethiopia; 2023.

Variables	Category	Frequency (*n*)	Percentage (%)
Perceived risk	Yes	160	68.1
	No	75	31.9
Perceived self-imagination	Yes	171	72.8
	No	64	27.2
Perceived need support	Yes	186	79.1
	No	49	20.9

### Perceived stigma and related characteristics

In this study, 118 (50.2%) anticipated experiencing stigma if they tested positive for HIV. Therefore, the overall perceived stigma was 50.2%. Almost half of the study participants (118, 50.2%) fear social rejection. Similarly, 132 (56.2%) of the study participants feel an internalized stigma and 101(43.0%) feel perceived discrimination. Consequently, 126 (53.6%) of the study participants feel lack confidentiality and 133 (56.6%) feel gender based stigma (Table 3).

**Table 3:** Self-perception and related characteristics of TB infected patients at public hospitals in Addis Ababa, Ethiopia; 2023.

Variables	Category	Frequency (*n*)	Percentage (%)
Fear of social rejection	No	117	49.8%
	Yes	118	50.2%
Internalized stigma	No	103	43.8%
	Yes	132	56.2%
Perceived discrimination	No	134	57.0%
	Yes	101	43.0%
Lack of confidentiality	No	109	46.4%
	Yes	126	53.6%
Gender based stigma	No	102	43.4%
	Yes	133	56.6%
Associations with promiscuity or drug use	No	106	45.1%
	Yes	129	54.9%
Cultural belief	No	112	47.7%
	Yes	123	52.3%
Impact on relationship	No	112	47.7%
	Yes	123	52.3%

### Knowledge on VCT

The majority of the study participants, 227 (96.6%), reported having information about VCT. In this study, 133 (56.6%) of the participants scored above the mean score in the knowledge assessment questions. Therefore, the proportion of TB-infected patients with good knowledge about VCT was 56.6% (95% CI: 50.2, 62.6). Among the respondents, 85 (36.2%) indicated that HIV testing is provided with counseling, and 26 (11.1%) stated that HIV testing is conducted voluntarily. Similarly, 32 (13.6%) of the participants were aware of the locations where VCT is provided, and 19 (8.1%) expressed the view that VCT is important for the prevention and control of HIV.

### Health facility and related factors

More than half of the study participants (123, 52.3%) travel for less than half an hour to reach the nearest health facility. Similarly, 135 (57.4%) of the study participants have less than 10 kilometers between their home and the nearest health facility. In addition, 95 (40.4%) of the study participants perceived that, their home is very near to the nearest health facility (Table 4).

**Table 4:** Health facility and related characteristics of TB infected patients at public hospitals in Addis Ababa, Ethiopia; 2023.

Variables	Category	Frequency (*n*)	Percentage (%)
Travel time to the nearest health facility	Less than half an hour	123	52.3%
	Half to 1 h	88	37.4%
	One to 2 h	20	8.5%
	More than 2 h	4	1.7%
Distance to the nearest health facility (in km)	Less than or equal to 10	135	57.4%
	More than 10	100	42.6%
Perceived distance to the nearby health facility	Very near	95	40.4%
	Near	48	20.4%
	Medium	32	13.6%
	Far	40	17.0%
	Very far	20	8.5%

### Willingness toward VCT

In this study, 184 (78.3%) of TB infected patients were willing toward VCT. Therefore, the prevalence of willingness toward VC among TB infected patients at Public Hospitals in Addis Ababa was 78.3% (95%CI, 72.8, 83.4).

### Factors associated with willingness toward VCT

The odd of the likelihood of having willingness toward VCT among TB infected patients who were studied primary education was six folds higher as compared with those who have no formal education (AOR: 6.32; 95%CI: 1.65, 24.25). Government employed TB patients were 5.85 times more likely to be willing toward VCT as compared with merchants (AOR: 5.85; 95%CI: 1.78, 19.22). Similarly, willingness toward VCT was 3.35 times higher among private employee TB infected patients as compared with merchants (AOR: 3.35; 95%CI: 1.12, 10.01). In addition, students were 3.71 times more likely to be willing toward VCT as compared with merchants (AOR: 3.71; 95%CI: 1.10, 12.49). The odd of the likelihood of having willingness toward VCT among patients who have poor knowledge on VCT was three folds higher as compared with the counterparts who have poor knowledge (AOR: 3.12;95%CI: 1.36, 7.16). TB infected patients who have perceived risk were6.58 times more likely to be willing toward VCT as compared with those who have no perceived risk (AOR: 6.58; 95%CI: 2.44, 17.73). In addition the odd of the likelihood of being willing toward VCT among TB infected patients who have perceived stigma was 15 times higher as compared with the counterparts who have no perceived stigma (AOR: 14.95; 4.98, 44.91) (Table 5).

**Table 5:** Factors associated with willingness toward VCT among TB infected patients at public hospitals in Addis Ababa, Ethiopia; 2023.

Variables	Category	Willingness toward VCT		COR (95%CI)	AOR (95%CI)
		Yes	No		
Sex	Male	141	32	1.95(1.01, 3.78)*	2.16(0.98, 4.75)
	Female	43	19	1	1
Level of education	No formal education	11	10	1	1
	Primary	44	10	4.0(1.34, 11.99)*	6.32(1.65, 24.25)**
	Secondary	54	10	4.91(1.65, 14.61)*	2.44(0.67, 8.87)
	College and above	75	21	3.25(1.21, 8.68)*	2.58(0.78, 8.56)
Occupation	Government employee	54	11	2.62(1.08, 6.36)*	5.85(1.78, 19.22)**
	Private employee	70	12	3.11(1.31, 7.37)*	3.35(1.12, 10.01)**
	Student	30	12	1.33(0.54, 3.29)	3.71(1.10, 12.49)**
	Merchant	30	16	1	1
Knowledge on VCT	Poor	70	32	1	1
	Good	114	19	2.74(1.45, 5.21)*	3.12(1.36, 7.16)**
Perceived risk	Yes	132	28	2.09(1.10, 3.95)*	6.58(2.44, 17.73)**
	No	52	23	1	1
Perceived stigma	Yes	79	39	4.32(2.12, 8.78)*	14.95(4.98, 44.91)**
	No	105	12	1	

* indicates variable having *P*-value < 0.25 in bivariate analysis and ** indicates variables having *P*-value < 0.05 in multivariable analysis.

## Discussion

The VCT stands out as a crucial tool in combatting the spread of HIV/AIDS and is recognized as a key element in HIV/AIDS prevention strategies (35–37). This study sought to assess the degree of willingness toward VCT among TB patients and explore the associated factors. Despite evidence from previous studies indicating low utilization of VCT services, especially in developing countries (38–41), this research aimed to delve into the levels of willingness and the factors influencing it among individuals with TB.

The study revealed that 56.6% (95%CI: 50.2, 62.6) of TB-infected patients possessed good knowledge about VCT. This finding aligns with a similar report from Ethiopia (42, 43). While the level of awareness may differ, reports from Nigeria and Tanzania have indicated a satisfactory level of knowledge regarding VCT (44–46). However, a community-based study in China identified a notable lack of knowledge about HIV and VCT (42), potentially attributed to variations in the socio-demographic characteristics, particularly educational levels, among the study participants.

The prevalence of willingness toward VCT among TB-infected patients at Public Hospitals in Addis Ababa was determined to be 78.3% (95%CI: 72.8, 83.4). This finding surpasses the results of a study conducted in Tanzania, where willingness stood at 30.3% (39). However, this proportion falls considerably short of the UNAIDS target, which aims for 90% of people to be aware of their HIV status. Numerous studies have highlighted the underutilization of VCT services in many developing countries, despite increased awareness of the benefits of testing (47, 48).

The level of education emerged as a significant factor associated with willingness toward VCT among TB patients. The odds of having willingness toward VCT among TB-infected patients who had received primary education were six times higher compared to those with no formal education (AOR: 6.32; 95%CI: 1.65, 24.25). Moreover, government-employed TB patients were 5.85 times more likely to express willingness toward VCT compared to merchants (AOR: 5.85; 95%CI: 1.78, 19.22). This finding aligns with a study in Zambia, suggesting that educated individuals, particularly women, may possess greater knowledge about VCT and understand the benefits of HIV testing (49). Additionally, results from other Sub-Saharan African countries have consistently shown that TB patients with a secondary education level or higher tend to be more knowledgeable about VCT (41, 50). These findings underscore the importance of education in promoting understanding and acceptance of VCT, as studies have indicated that insufficient awareness about VCT contributes to the reluctance of TB patients to undergo HIV testing (40, 41, 51).

Occupation was identified as a significant factor associated with willingness toward VCT among TB patients. TB-infected patients employed in the private sector were 3.35 times more likely to express willingness toward VCT compared to merchants (AOR: 3.35; 95%CI: 1.12, 10.01). Moreover, students demonstrated a 3.71 times higher likelihood of being willing toward VCT compared to merchants (AOR: 3.71; 95%CI: 1.10, 12.49). This association may be attributed to mandatory testing during employment, which could prompt job applicants to seek knowledge about their HIV status or those with the potential to be diagnosed as HIV positive (52). Additionally, employees and students might have better access to information about HIV, encouraging TB-infected patients to be more willing to undergo VCT.

Knowledge was identified as a significant factor associated with willingness toward VCT. The likelihood of being willing toward VCT among patients with poor knowledge on VCT was three times higher compared to those with good knowledge (AOR: 3.12; 95%CI: 1.36, 7.16). This finding aligns with a study conducted in North West Ethiopia (53). The result supports the notion that individuals seeking VCT may have greater exposure, information, and knowledge about HIV/AIDS before visiting VCT centers (15). This underscores the importance of health education and counseling sessions in disseminating information, suggesting that including relevant topics in these sessions and maintaining continuous mass media activity could enhance knowledge and willingness toward VCT.

Perceived risk was identified as a significant factor associated with willingness toward VCT among TB patients. TB-infected patients who perceived a risk were 6.58 times more likely to be willing toward VCT compared to those who perceived no risk (AOR: 6.58; 95%CI: 2.44, 17.73). This finding is consistent with a study conducted in North West Ethiopia (53). It emphasizes the importance of individuals’ perception of their own risk as a motivating factor for their willingness to undergo VCT, suggesting that interventions should address and enhance individuals’ awareness of the potential risks associated with HIV.

The likelihood of being willing toward VCT among TB-infected patients who perceived stigma was 15 times higher compared to those who had no perceived stigma (AOR: 14.95; 4.98, 44.91). This finding aligns with a study conducted at the National Hospital Ambulatory Medical Care (54). The reluctance to undergo HIV testing may be linked to the potential consequences, such as loss of relationships, employment, housing, and other forms of stigmatization upon discovering a positive result. This fear of stigma can act as a significant barrier to HIV testing (55, 56).

### Limitation of the study

It’s important to note that this study’s cross-sectional design does not establish a cause-and-effect relationship, and being a facility-based study limits its generalizability to the broader community. Consistently, this study did not assess HIV serostatus result of the study participants and their CD4 cell count.

## Conclusion

Despite a higher proportion of TB-infected patients expressing willingness toward VCT, the rates fall below the UNAIDS target of 90% of people knowing their HIV status. Factors such as level of education, occupation, knowledge, perceived risk, and perceived stigma were identified as independent factors associated with willingness toward VCT. To meet WHO standards for HIV testing, it is crucial for the government to implement strategies aimed at providing information, especially to those with lower levels of education. These efforts can contribute to achieving higher rates of awareness and acceptance of VCT services among TB patients.

## Data availability statement

The original contributions presented in the study are included in the article/supplementary materials, further inquiries can be directed to the corresponding author.

## Ethics statement

The studies involving humans were approved by Yekatit 12 Hospital Medical College Ethical Review Board. The studies were conducted in accordance with the local legislation and institutional requirements. The ethics committee/institutional review board waived the requirement of written informed consent for participation from the participants or the participants’ legal guardians/next of kin because it was verbal consent to encompass the illiterates.

## Author contributions

SS: Conceptualization, Data curation, Formal analysis, Funding acquisition, Investigation, Methodology, Project administration, Resources, Software, Supervision, Validation, Visualization, Writing – original draft, Writing – review & editing. MG: Conceptualization, Data curation, Formal analysis, Funding acquisition, Investigation, Methodology, Project administration, Resources, Software, Supervision, Validation, Visualization, Writing – review & editing.
